# Olfactory Stimulation Selectively Modulates the OFF Pathway in the Retina of Zebrafish

**DOI:** 10.1016/j.neuron.2013.05.001

**Published:** 2013-07-10

**Authors:** Federico Esposti, Jamie Johnston, Juliana M. Rosa, Kin-Mei Leung, Leon Lagnado

**Affiliations:** 1 Laboratory of Molecular Biology, Medical Research Council, Cambridge CB2 0QH, UK; 2 School of Life Sciences, University of Sussex, Falmer, Brighton BN1 9QG, UK

## Abstract

Cross-modal regulation of visual performance by olfactory stimuli begins in the retina, where dopaminergic interneurons receive projections from the olfactory bulb. However, we do not understand how olfactory stimuli alter the processing of visual signals within the retina. We investigated this question by in vivo imaging activity in transgenic zebrafish expressing SyGCaMP2 in bipolar cell terminals and GCaMP3.5 in ganglion cells. The food-related amino acid methionine reduced the gain and increased sensitivity of responses to luminance and contrast transmitted through OFF bipolar cells but not ON. The effects of olfactory stimulus were blocked by inhibiting dopamine uptake and release. Activation of dopamine receptors increased the gain of synaptic transmission in vivo and potentiated synaptic calcium currents in isolated bipolar cells. These results indicate that olfactory stimuli alter the sensitivity of the retina through the dopaminergic regulation of presynaptic calcium channels that control the gain of synaptic transmission through OFF bipolar cells.

## Highlights

• Olfactory stimuli regulate transmission of signals through retinal bipolar cells
• Modulation of synaptic gain and sensitivity occur in OFF bipolar cells but not ON
• An inhibitor of dopamine uptake blocks odor-induced changes in synaptic gain
• Dopamine potentiates presynaptic calcium channels in isolated bipolar cells

## Teaser

Esposti et al. show that olfactory stimulation selectively modulates synaptic transmission from retinal OFF bipolar cells in zebrafish. Dopamine plays a key role in this cross modal interaction by acting on the presynaptic calcium channels.

## Introduction

The vertebrate retina receives efferent inputs from different parts of the central nervous system but we still do not understand how these regulate visual processing (Ramon y Cajal, 1894, Repérant et al., 1989). In teleosts, the main source of retinopetal fibers is the terminal nerve (TN), which receives dense afferents from the olfactory bulb and in turn projects GnRH- and FMRFamide-containing fibers to the retina (Springer, 1983, Zucker and Dowling, 1987, Demski, 1993, Yamamoto and Ito, 2000, Repérant et al., 2007). The TN is tonically active, with a firing frequency that changes according to the physiological conditions of the animal, including arousal, motivational state, hormonal milieu, and glutamatergic inputs from various sensory systems (Abe and Oka, 2006, Wang et al., 2011). Together, the pathways linking the olfactory bulb to the retina through the TN are known as the olfacto-retinal circuit (ORC). Behavioral assays examining visual threshold have shown that stimulation of the olfactory bulb by food-related amino acids induces an increase in luminance sensitivity through activation of the ORC (Maaswinkel and Li, 2003, Behrens and Wagner, 2004, Li and Maaswinkel, 2007).

It has been suggested that an olfactory stimulus alters the processing of visual signals by decreasing the concentration of dopamine in the retina (Huang et al., 2005). The sole source of dopamine in the retina of teleosts is a specialized class of amacrine cell, the interplexiform cells (IPCs), which are the target of the TN (Umino and Dowling, 1991). Li and Dowling (2000a) have shown that zebrafish affected by the night blindness b mutation (*nbb*), which provokes a progressive reduction in the number of IPCs, exhibit a 2–3 log unit decrease in luminance sensitivity and a profound loss of signals derived from rods. Dopamine (DA) released from IPCs has a number of actions on the retinal circuit, which together act to enhance cone-mediated signals under bright conditions. In the outer retina, dopamine decreases electrical coupling between rods and cones (Ribelayga et al., 2008), while inhibiting voltage-gated calcium currents in rods and boosting calcium currents in cones (Stella and Thoreson, 2000). Dopamine also inhibits electrical coupling between horizontal cells and increases their sensitivity to glutamate, resulting in less powerful negative feedback to cones (Knapp and Dowling, 1987, DeVries and Schwartz, 1989, McMahon, 1994). In the inner retina, dopamine modulates electrical coupling between amacrine cells (Feigenspan and Bormann, 1994). Actions on bipolar cells and retinal ganglion cells (RGCs) have also been reported, but their roles in altering retinal processing under different lighting conditions are not clearly established (Jensen and Daw, 1984, Jensen, 1992, Heidelberger and Matthews, 1994, Li and Dowling, 2000b, Ribelayga et al., 2002).

How might the actions of dopamine underlie the modulation of retinal processing by an olfactory stimulus? One of the difficulties in studying a multisensory circuit is the need to conduct experiments in vivo in order to maintain the link between the different sensory systems. In this study, we take advantage of zebrafish expressing genetically encoded calcium reporters in the synaptic terminals of bipolar cells or dendrites of RGCs (Dreosti et al., 2009, Odermatt et al., 2012). These fish allow the visual signal to be monitored as it is transmitted to the inner retina and RGCs providing the output from this circuit. By imaging signals through all layers of the inner retina, we have observed activity at the origins of the ON and OFF channels that encode a change in light intensity with signals of opposite polarity (Schiller et al., 1986).

Here, we demonstrate that an olfactory stimulus reduces the gain but increases the sensitivity with which OFF bipolar cells transmit signals encoding luminance and contrast. No effect could be detected on the large majority of ON bipolar cells. Pharmacological manipulations in vivo demonstrated that olfactory stimuli regulate the presynaptic calcium signal of bipolar cells by reducing the activity of D_1_ dopamine receptors, and electrophysiology in isolated bipolar cells demonstrated that activation of endogenous dopamine receptors potentiates voltage-dependent calcium channels that control synaptic transmission. Together, these results indicate that the ORC acts through the neuromodulator dopamine to regulate synaptic transmission through the OFF channel in the retina.

## Results

### Olfactory Stimulation Modulates Luminance Signaling through OFF Bipolar Cells

**Figure 1:**
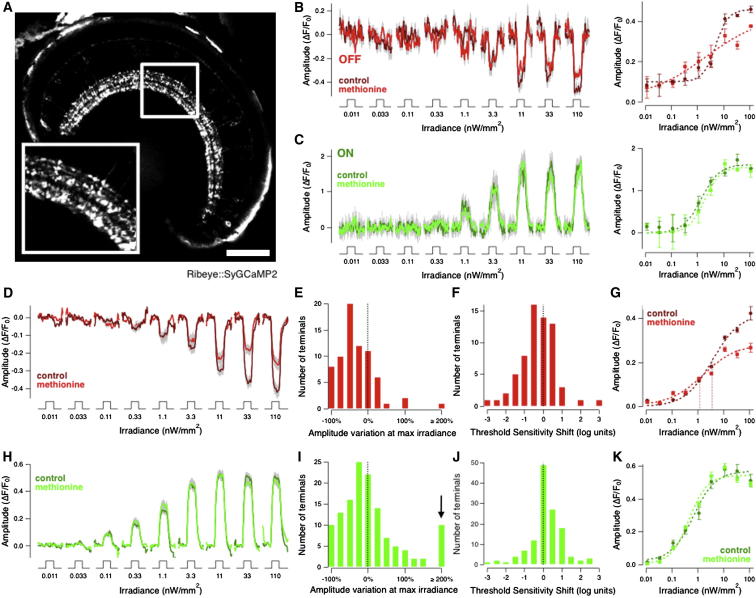
Olfactory Stimulation Selectively Reduces the Gain and Increases the Sensitivity of Luminance Responses through the OFF Visual Pathway (A) Retina of a ribeye::SyGCaMP2 fish showing synaptic terminals of bipolar cells in the IPL. Scale bar represents 100 μm. Inset shows 2× expansion. (B) Responses of an individual OFF bipolar cell terminal to 3 s steps of light increasing in irradiance (i.e., light intensity) by 0.5 log unit steps. Control responses in dark red and responses in methionine in light red. Average of four repetitions; SEM indicated in gray. Corresponding plot of intensity versus response amplitude to the right. Dotted lines are fits of the Hill function (see Experimental Procedures), with *h* = 1.77 and *I*_*1/2*_ = 4.94 nW/mm^2^ in control and *h* = 0.39, *I*_*1/2*_ = 2.10 nW/mm^2^ in methionine. (C) Responses of an individual ON bipolar cell terminal to the same series of stimuli shown in (B). Control responses dark green and responses in methionine in light green. Corresponding plot of intensity versus response amplitude to the right. No significant difference on application of methionine (*h* = 1.24 and *I*_*1/2*_ = 1.40 nW/mm^2^ in control and *h* = 1.45, *I*_*1/2*_ = 1.83 nW/mm^2^ in methionine). (D) Averaged responses of 84 OFF bipolar cell terminals from seven fish (8–11 dpf) to stimulus protocol shown in (B). Average amplitude of response to bright light was reduced by 36.6% ± 8.2%. SEM is shown in gray. (E) Histogram showing the percentage changes in the SyGCaMP2 responses of OFF terminals from (D), after application of methionine (response to the brightest step of light, 110 nW/mm^2^). (F) Histogram showing the threshold sensitivity shifts in the SyGCaMP2 responses to light of OFF terminals from (D), after application of methionine. The median increase in sensitivity was −0.5 log units, significantly different from 0 (p = 0.02). (G) Intensity versus response amplitude plot averaged from the same population of OFF terminals shown in (D). Dotted lines are fits of the Hill function with *h* = 0.70 and *I*_*1/2*_ = 6.16 nW/mm^2^ in control and *h* = 0.66, *I*_*1/2*_ = 1.86 nW/mm^2^ in methionine. Note that methionine application increased sensitivity by lowering *I*_*1/2*_ by 0.52 log units, i.e., 2.75 times (p < 0.0001). (H) Averaged responses of 116 ON bipolar cell terminals from seven fish (8–11 dpf) to stimulus protocol shown in (B). Across this complete population, methionine did not cause any significant change in luminance response. (I) Histogram showing the percentage changes in the SyGCaMP2 responses of ON terminals from (H), after application of methionine (response to the brightest step of light, 110 nW/mm^2^). Average increase was 5% ± 7% (average ± SEM). Note the small population of terminals (∼9%) in which methionine caused an increase in the SyGCaMP2 response to bright light (arrow; 3-fold or more). See also Figures S1A and S1B. (J) Histogram showing the sensitivity shifts in the SyGCaMP2 responses to light of ON terminals from (H), after application of methionine. The median shift in sensitivity was 0. (K) Intensity versus response amplitude plot averaged from the same population of ON terminals shown in (H). Dotted lines are fits of the Hill function with *h* = 0.93 and *I*_*1/2*_ = 0.84 nW/mm^2^ in control and *h* = 1.14, *I*_*1/2*_ = 0.60 nW/mm^2^ in methionine. All responses were recorded between 9:00 and 11:00 a.m. For early afternoon experiments, please see Figures S1C–S1F. See also Figure S1.

Transmission of the visual signal through the ON and OFF pathways in the retina was assessed in transgenic zebrafish expressing SyGCaMP2 under the ribeye promoter (Dreosti et al., 2009), allowing synaptic activity to be monitored across the population of bipolar cells projecting to all layers of the inner plexiform layer (IPL) (Figure 1A). First, we investigated the effects of olfactory stimulation on the response to changes in the luminance of full-field stimuli. All measurements were carried out between 9:00 and 11:00 a.m., when behavioral experiments have demonstrated that the ORC is most effective in modulating visual sensitivity (Maaswinkel and Li, 2003). Figure 1B shows responses of an individual OFF terminal to light steps of different intensity, before (dark red) and after (light red) the addition of 1 mM methionine to the medium surrounding the fish. In OFF terminals, there was a decrease in the maximum amplitude of the response, which we term a decrease in gain. Additionally, OFF terminals began to respond at lower intensities, which we term an increase in sensitivity. In contrast, the large majority of ON bipolar cells were unaffected by olfactory stimulation, and an individual example is shown in Figure 1C.

Collected results from 84 OFF terminals from seven fish are shown in Figures 1D–1G. Methionine reduced the response to the brightest step of light by an average of 36.6% ± 8.2% (p < 0.0001), and this effect was evident across the population of OFF terminals (Figure 1E). In parallel (Figure 1F), methionine increased the luminance sensitivity of OFF terminals by 0.5 log units, assessed by the lowest light level eliciting responses >3 SD of the baseline noise. A similar shift could be assessed by fitting the intensity-response measurements with a Hill function (Figure 1G) and estimating *I*_*1/2*_, the intensity generating a half-maximal response (Experimental Procedures). The increase in luminance sensitivity observed at the synaptic output of bipolar cells was quantitatively similar to the increase observed previously in ganglion cell recordings and behaviorally following olfactory stimulation (Maaswinkel and Li, 2003, Huang et al., 2005).

The actions of methionine were almost exclusively on the OFF pathway. The large majority of ON synapses in a population of 116 were not affected, either in terms of response amplitude or sensitivity (Figures 1H–1K). The exception was a small but distinct fraction of terminals (∼9%), in which the amplitude of the presynaptic calcium signal was increased by a factor of at least three (arrow in Figure 1I and described further in Figures S1A and S1B available online). This subset of ON terminals was not distinguishable from others in terms of size or position in the IPL and is therefore unlikely to reflect a difference between cone-driven and mixed rod-cone bipolar cells. No significant changes in ON or OFF responses were observed over the same time window in the absence of methionine. Although behavioral experiments showed olfactory stimulation to be ineffective in the afternoon (Maaswinkel and Li, 2003), we found that methionine administration produced a qualitatively similar but significantly smaller modulation of responses through the OFF pathway tested between 12:00 and 3:30 p.m. (Figures S1C–S1F).

### Olfactory Stimulation Depresses the Transmission of Contrast Variations through OFF Bipolar Cells

**Figure 2:**
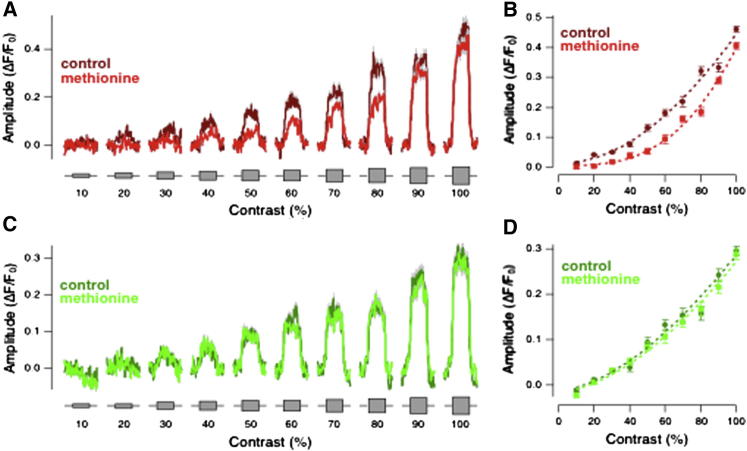
Olfactory Stimulation Reduces Synaptic Responses to Variations in Temporal Contrast in OFF Bipolar Cells (A) Averaged responses of 45 OFF bipolar cell terminals from five fish to contrasts between 10% and 100% (5 Hz, square wave, mean intensity 55 nW/mm^2^). Control responses in dark red and responses in methionine in light red. SEM indicated in gray. (B) Plot of contrast versus response amplitude averaged from the same population of OFF terminals shown in (A). Note that the response is measured as the steady deflection from the baseline. Dotted lines are fits of a power function, *A* = *k* × *C*^*α*^, with α = 1.81 ± 0.12 in control and α = 2.87 ± 0.15 in methionine. (C) Averaged responses of 44 ON bipolar cell terminals from five fish to the same temporal contrast protocol in (A). Control responses in dark green, and responses in methionine in light green. SEM indicated in gray. See also Figure S2. (D) Plot of response amplitude versus contrast averaged from the same population of ON terminals shown in (C). Dotted lines are fits of a power function with α = 1.66 in control and α = 1.65 in methionine. Methionine did not significantly alter the synaptic response of ON bipolar cells. See also Figure S2.

Many retinal neurons signal fluctuations in light intensity at frequencies up to ∼20 Hz. To test the effects of an olfactory stimulus on the signaling of temporal contrast, we modulated full-field stimuli at 5 Hz and measured the SyGCaMP2 signal (5 fish, n = 122 terminals). Figure 2 shows that the most obvious effect of an increase in contrast was a steady offset in the SyGCaMP2 signal, reflecting a net accumulation of calcium. A change in the SyGCaMP2 signal in the absence of a change in the mean luminance demonstrates a strong rectification in the bipolar cell terminal. The relation between the amplitude of the steady SyGCaMP2 signal (*A*) and contrast (*C*) is shown in Figure 2B: it could be described by a simple power function of the form *A* = *k* × *C*^*α*^, with α = 1.8 ± 0.1 under control conditions. Stimulation with methionine reduced the amplitude of the rectifying response at all contrasts above 20% (Figure 2A). Furthermore, methionine increased the exponent α of the power function to 2.9 ± 0.2 (p < 0.0001). This renders OFF terminals more sensitive to higher contrasts at the expense of lower contrasts, e.g., a change in temporal contrast from 80% to 90% caused a change of 23.5% ΔF/F_0_ in control versus 40% ΔF/F_0_ in methionine. Olfactory stimulation did not, however, affect the responses of ON bipolar cells (Figures 2C and 2D). We also observed a small subset of ON bipolar cells in which the DC presynaptic calcium levels were reduced by stimulation at 5 Hz, and these were also unaffected by application of methionine (Figures S2A and S2B).

The inhibition of presynaptic calcium signals by olfactory stimulation was also a function of stimulus frequency. We tested the responses of OFF terminals to frequencies between 0.2 and 25 Hz at 90% contrast (6 fish, n = 96 terminals) and found that methionine reduced the amplitude of the SyGCaMP2 signal elicited by stimuli below 10 Hz. Again this effect was specific to the OFF pathway (Figures S2C–S2H).

Together, the results in Figures 1, 2, and S2 indicate that olfactory stimulation alters the processing of visual signals in the retina by two distinct actions on OFF bipolar cells: a suppression of the presynaptic calcium signal, resulting in a reduction in gain and an increase in luminance sensitivity. The large majority of ON bipolar cells were unaffected by this form of cross-modal regulation.

### Olfactory Stimulation Depresses Signaling through OFF Ganglion Cells

**Figure 3:**
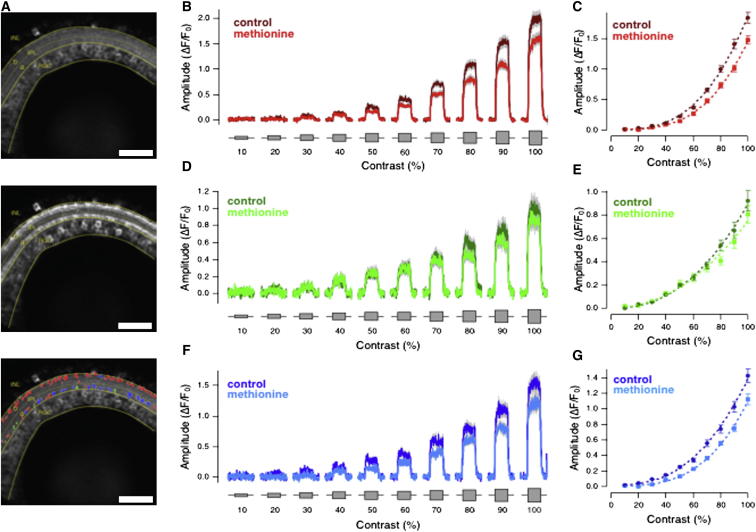
Olfactory Stimulation Reduces Synaptic Responses to Variations in Temporal Contrast in OFF and ON-OFF Retinal Ganglion Cell Dendrites (A) Example of a eno2::GCaMP3.5 fish retina, recorded in vivo, at rest (top), and during contrast stimulation (middle). The panel on the bottom shows the responding regions of the IPL color-coded according to their polarity (OFF, red; ON, green; ON-OFF, blue; see also Figure S3). Please note the calcium reporter expression in the IPL and in the RGC layer, plus some rare displaced retinal ganglion cells localized in the inner nuclear layer (INL). Note also the preferential distribution of OFF responses in the *sublamina b* of the IPL and of ON and ON-OFF responses in the *sublamina a* of the IPL (bottom). Scale bar represents 200 μm. (B) Average response of 186 OFF ROIs from RGC dendrites n = 5 eno2::GCaMP3.5 transgenic zebrafish to the same temporal contrast protocol in Figure 2, before (dark red) and after (light red) methionine administration. SEM is indicated in gray. Please note how methionine reduced the amplitude of OFF responses in a similar way to that observed for OFF bipolar cell terminals (Figure 2A). (C) Plot of contrast versus response amplitude averaged from the same population of OFF ROIs shown in (B). As for Figure 2, the response is measured as the steady deflection from the baseline. Dotted lines are fits of a power function, *A* = *k* × *C*^*α*^, with α = 2.96 ± 0.11 in control and α = 3.27 ± 0.09 in methionine. (D and E) Average response to contrast and contrast versus response amplitude plot of 69 ON ROIs from RGC dendrites, to the same temporal contrast protocol in Figure 2, before (dark green) and after (light green) methionine administration. Dotted lines are fits of a power function, *A* = *k* × *C*^*α*^, with α = 2.32 ± 0.12 in control and α = 2.11 ± 0.20 in methionine. (F and G) Average response to contrast and contrast versus response amplitude plot of 79 ON-OFF ROIs from RGC dendrites, to the same temporal contrast protocol in Figure 2, before (dark blue) and after (light blue) methionine administration. Dotted lines are fits of power functions, *A* = *k* × *C*^*α*^, with α = 2.65 ± 0.25 in control and α = 3.18 ± 0.15 in methionine. Please note that methionine reduced the amplitude of ON-OFF responses in a similar way to that observed for OFF retinal ganglion cells (B and C) and OFF bipolar cell terminals (Figures 2A and 2B). See also Figure S3.

Having observed an action of olfactory stimulation on the visual signal as it is transmitted by bipolar cells, we investigated how far the responses of postsynaptic ganglion cells were also affected. To monitor signals across large populations of neurons in vivo, we made a line of zebrafish expressing the calcium reporter GCaMP3.5 under the eno2 promoter, which drives expression in RGCs (Bai et al., 2007; Figure 3A). Responses were then quantified in RGC dendrites through different strata of the IPL. Step changes in luminance were a relatively ineffective stimulus for RGCs, so we examined the effects of an olfactory stimulus on full-field stimuli modulated at 5 Hz. The advantage of this in vivo imaging approach over electrophysiology is that it allows stimulation of the olfactory system while observing activity across a large population of RGCs.

Responses from RGC dendrites were classified as OFF (Figures 3B and 3C), ON (Figures 3D and 3E), or ON-OFF (Figures 3F and 3G), according to the responses to steps of light (Figure S3). A total of 334 responses from n = 5 eno2::GCaMP3.5 fish were collected. Methionine induced a reduction in gain of OFF and ON-OFF RGCs at contrasts of 50% and above, without an appreciable effect on ON RGCs. These results are consistent with the reduced gain of responses to contrast observed in OFF bipolar cell terminals, but not ON, following application of methionine (Figure 2). They also confirm that the actions of the ORC are evident in the retinal output, as previously demonstrated by Maaswinkel and Li (2003) and Huang et al. (2005).

### D_1_ Dopamine Receptors Are Involved in Olfactory Modulation of Retinal Function

How does an olfactory stimulus modulate synaptic transmission through bipolar cells? Existing evidence suggests that a key signal is dopamine released by IPCs (Umino and Dowling, 1991, Huang et al., 2005). To investigate how dopaminergic signaling might be involved in modulating synaptic activity of bipolar cells, we injected agonists or antagonists of dopamine receptors into the anterior chamber of one eye of a fish, with a parallel sham injection into the other eye acting as a control.

**Figure 4:**
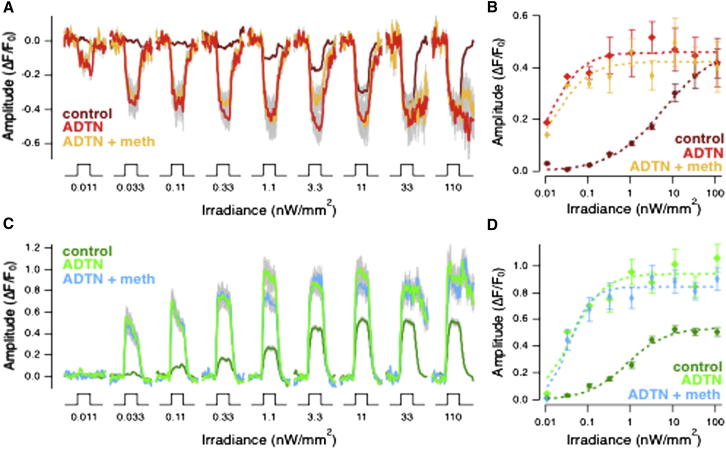
The Dopamine Receptor Agonist ADTN Increases the Gain of Synaptic Responses in Bipolar Cells and Blocks Modulation by an Olfactory Stimulus (A) Averaged responses of 28 OFF bipolar cell terminals from four fish injected intraocularly with ADTN (light red; 0.2 μM estimated final concentration). The stimuli are a series of light steps of increasing intensity, as in Figure 1. Control responses (dark red) are from noninjected fish (traces in Figure 1D). The yellow traces show responses measured when methionine was applied in the presence of ADTN. SEM is shown in gray. (B) Plot of intensity versus response amplitude averaged from the same populations of OFF terminals shown in (A). Dotted lines are fits of the Hill function with *h* = 0.70 and *I*_*1*/2_ = 6.16 nW/mm^2^ in control, *h* = 1.15 and *I*_*1/2*_ = 0.01 nW/mm^2^ in ADTN before olfactory stimulation and *h* = 1.04 and *I*_*1/2*_ = 0.02 nW/mm^2^ in ADTN after olfactory stimulation. Note that ADTN increased luminance sensitivity of OFF bipolar cell terminals by 2.79 log units, but did not significantly affect the amplitude of the response to brightest lights. Methionine did not significantly alter sensitivity or maximum response (yellow). (C) Averaged responses of 64 ON bipolar cell terminals from four fish collected simultaneously with results shown in (A) and (B). Control responses (dark green) correspond to traces in Figure 1H. The light blue traces show responses measured when methionine was applied in the presence of ADTN. (D) Plot of intensity versus response amplitude averaged from the same populations of ON terminals shown in (C). Dotted lines are fits of the Hill function with *h* = 0.93 and *I*_*1/2*_ = 0.84 nW/mm^2^ in control, *h* = 1.16 and *I*_*1/2*_ = 0.04 nW/mm^2^ in ADTN before olfactory stimulation and *h* = 1.65 and *I*_*1/2*_ = 0.04 nW/mm^2^ in ADTN after olfactory stimulation. Note that ADTN increased luminance sensitivity of ON bipolar cell terminals by 1.32 log units and also doubled the maximum amplitude of the response to the brightest lights. Methionine did not significantly alter sensitivity or maximum response in the presence of ADTN (light blue).

The first manipulation was to activate dopamine receptors by injecting the agonist [3H] 2-amino-6,7-dihydroxy 1,2,3,4-tetrahydronapthalene (ADTN) at an estimated concentration of 0.2 μM (see Experimental Procedures). In OFF terminals, ADTN increased the amplitude of SyGCaMP2 responses to all but the brightest lights and luminance sensitivity (*I*_*1/2*_) increased by a factor of ∼420 (Figures 4A and 4B; n = 92 terminals). In ON terminals, ADTN increased the amplitude of the SyGCaMP2 response to bright lights by 108% and increased luminance sensitivity by a factor of 15 (Figures 4C and 4D). Strong activation of dopamine receptors therefore potentiated presynaptic calcium signals in both ON and OFF bipolar cells, an effect opposite to an olfactory stimulus. Further, application of methionine in the presence of ∼0.2 μM ADTN no longer depressed signals through OFF bipolar cells (Figures 4A and 4B). Both these observations are consistent with the idea that an olfactory stimulus modulates retinal function by decreasing dopamine release.

**Figure 5:**
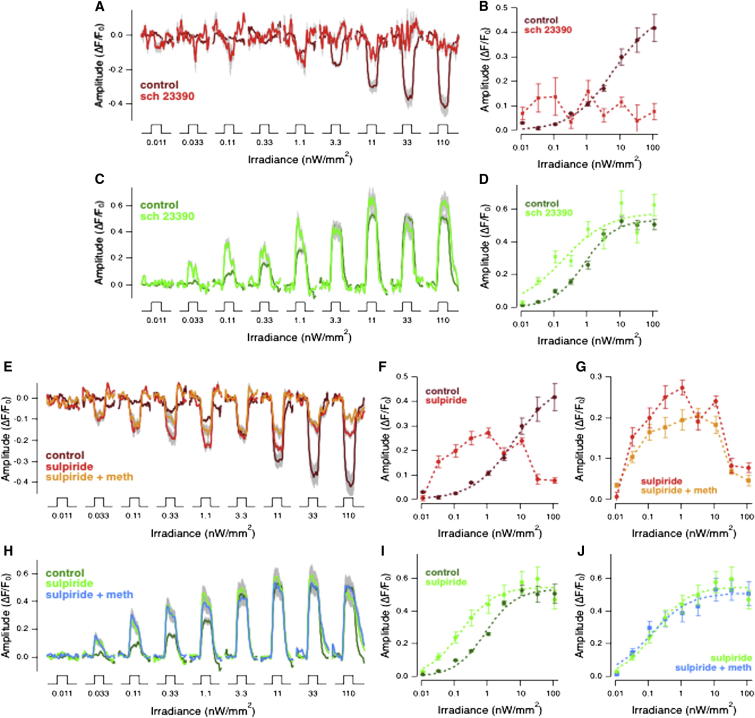
The Effect of Olfactory Stimulation Is Mirrored by a D_1_ but Not a D_2_ Dopamine Receptor Antagonist (A) Averaged responses of 17 OFF bipolar cell terminals from three fish injected intraocularly with the dopamine D_1_ receptor antagonist SCH 23390 (100 nM; light red). The stimuli are a series of light steps of increasing intensity, as in Figure 1. Control responses are from Figure 1D (dark red). SEM is shown in gray. (B) Plot of intensity versus response amplitude averaged from the same population of OFF terminals shown in (A). Antagonizing D_1_ receptors profoundly suppressed transmission of visual signals through synapses of OFF bipolar cells. (C) Averaged responses of 20 ON bipolar cell terminals from three fish collected simultaneously with results shown in (A). Control responses (dark green) correspond to traces in Figure 1H. The light green traces show responses measured after SCH 23390 100 nM injection. (D) Plot of intensity versus response amplitude averaged from the same populations of ON terminals shown in (C). Dotted lines are fits of the Hill function with *h* = 0.93 and *I*_*1/2*_ = 0.84 nW/mm^2^ in control and *h* = 0.62 and *I*_*1/2*_ = 0.19 nW/mm^2^ in SCH 23390 100 nM. SCH 23390 increased luminance sensitivity by 0.65 log units without significantly altering the amplitude of responses to the brightest lights. (E) Averaged responses of 45 OFF bipolar cell terminals from three fish injected intraocularly with the dopamine D_2_ receptor antagonist sulpiride (2 μM), before (light red) and after (orange) bath administration of methionine 1 mM. The stimuli are a series of light steps of increasing intensity, as in Figure 1. Control responses are from Figure 1D (dark red). SEM is shown in gray. (F and G) Plot of intensity versus response amplitude averaged from the same population of OFF terminals shown in (E). Control responses (dark red) are from Figure 1D. Please note how sulpiride injection altered the shape of the sensitivity curve of OFF terminals (F), without interfering with the effect of methionine administration (cf. E and G with Figures 1D and 1G). (H) Averaged responses of 84 ON bipolar cell terminals from three fish after intraocular injection of 2 μM sulpiride, before (light green) and after (light blue) bath administration of methionine. Control responses (dark green) correspond to traces in Figure 1H. (I and J) Plot of intensity versus response amplitude averaged from the same populations of ON terminals shown in (H). Dotted lines are fits of the Hill function with *h* = 0.93 and *I*_*1/2*_ = 0.84 nW/mm^2^ in control, *h* = 0.87 and *I*_*1/2*_ = 0.15 nW/mm^2^ in sulpiride, and *h* = 0.74 and *I*_*1/2*_ = 0.12 nW/mm^2^ in sulpiride and methionine. Sulpiride increased luminance sensitivity by 0.85 log units as compared to the control trace without significantly altering the amplitude of responses to the brightest lights. No significant effect of methionine administration was observable in this condition.

The second manipulation was to antagonize the action of endogenous dopamine by injection of 100 nM SCH 23390 (7-chloro-3-methyl-1-phenyl-1,2,4,5-tetrahydro-3-benzazepin-8-ol), a selective antagonist of dopamine D_1_ receptors (Mora-Ferrer and Neumeyer, 1996, Bourne, 2001). SCH 23390 injection resulted in a complete impairment of luminance signaling through OFF bipolar cells (Figures 5A and 5B). In contrast, the maximum amplitude of the response in ON bipolar cells was not significantly affected, although the light sensitivity (*I*_*1/2*_) was increased by a factor of 3.8 (Figures 5C and 5D). Antagonizing D_1_ receptors therefore caused effects qualitatively similar to an olfactory stimulus: a selective decrease in the gain of signaling through the OFF pathway (cf. Figures 1 and 5).

To investigate the role of D_2_ receptors, we used the antagonist sulpiride at a concentration of ∼2 μM (Lin and Yazulla, 1994, Mora-Ferrer and Gangluff, 2000). Sulpiride altered the luminance-response function in two ways. First, the sensitivity increased sufficiently to reduce threshold by ∼2 log units, likely reflecting the potentiation of rod inputs (Ribelayga et al., 2008). Second, the luminance-response relation did not simply rise monotonically, but instead passed through a maximum (Figures 5E and 5F). Despite these changes in circuit function, the maximum response to luminance was reduced by 29% ± 5.6% when methionine was applied after injection of sulpiride, an effect similar to that of olfactory stimulation under control conditions (Figures 5E and 5G). Sulpiride also increased sensitivity of signals through ON bipolar cells (0.85 log units), but methionine had no effect on the amplitude of these responses (Figures 5H–5J). An olfactory stimulus therefore continued to cause a selective reduction of responses through the OFF pathway when activation of D_2_ receptors was blocked. Together, the results in Figures 4 and 5 indicate that cross-modal regulation of retinal function depends primarily on the activity of D_1_ receptors.

### An Inhibitor of Dopamine Uptake Blocks Olfactory Modulation of Retinal Function

To test further the idea that activation of the ORC acts by decreasing dopamine level in the retina, we attempted to prevent these changes without interfering with the activity of dopamine receptors. Our strategy was to inject vanoxerine (GBR 12909; 2 μM), a potent and specific blocker of the transporters involved in dopamine reuptake from extracellular space and into secretory vesicles (Reith et al., 1994, Singh, 2000). Vanoxerine administration has been reported to result in a small but steady increase in extracellular dopamine concentration, followed by a persistent “clamp” once both uptake and release are blocked (Rothman et al., 1991, Lima et al., 1994, Reith et al., 1994, Schlicker et al., 1996, Kubrusly et al., 2008).

**Figure 6:**
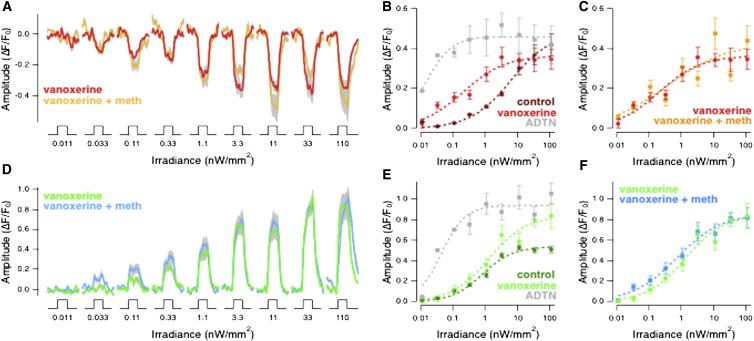
Vanoxerine, a Blocker of Dopamine Release and Reuptake, Prevents Olfactory Modulation of Signal Transmission from Bipolar Cells (A) Averaged responses of 37 OFF bipolar cell terminals from 4 fish injected intraocularly with vanoxerine. The stimuli are a series of light steps of increasing intensity, as in Figure 1. Vanoxerine-injected fish before olfactory stimulation in light red, and after olfactory stimulation in orange. SEM is shown in gray. (B and C) Plots of intensity versus response amplitude averaged from the same populations of OFF terminals shown in (A). Control responses (dark red) are from Figure 1D (*h* = 0.70 and *I*_*1/2*_ = 6.16 nW/mm^2^). Responses after vanoxerine injection in orange (*h* = 0.67 and *I*_*1/2*_ = 0.21 nW/mm^2^). Responses in vanoxerine after olfactory stimulation in yellow (*h* = 0.46 and *I*_*1/2*_ = 0.39 nW/mm^2^): note the lack of any significant difference. As a comparison, (B) also shows the intensity versus response amplitude plot for ADTN, as in Figure 4B (light gray). (D) Averaged responses of 32 ON bipolar cell terminals from 4 fish injected intraocularly with vanoxerine collected simultaneously with results in (A). Vanoxerine-injected fish before olfactory stimulation in light green and after olfactory stimulation in light blue. SEM indicated in gray. (E and F) Plots of intensity versus response amplitude averaged from the same populations of ON terminals shown in (D). Control responses (dark green) from Figure 1H. Responses after vanoxerine injection in light green (*h* = 0.74 and *I*_*1/2*_ = 1.34 nW/mm^2^). Responses after addition of methionine in the presence of vanoxerine in light blue (*h* = 0.63 and *I*_*1/2*_ = 0.75 nW/mm^2^). As a comparison, (E) also shows the intensity versus response amplitude plot for ADTN, as in Figure 4D (light gray). No statistically significant effect of methionine administration was observed.

Intraocular injection of 2 μM vanoxerine had two effects. First, the luminance sensitivity of OFF terminals was increased by a factor of 26 (Figures 6A and 6B) and of ON terminals by a factor of ∼2 (Figures 6D and 6E). Notably, these increases in sensitivity were much smaller than those caused by the dopamine receptor agonist ADTN (Figures 6B and 6E), indicating that increases in dopamine levels were relatively small and not sufficient to saturate dopamine receptors. The second action of vanoxerine was to prevent the application of methionine from modulating luminance signaling through OFF bipolar cells (Figures 6A and 6C), consistent with the idea that this modulation occurs through changes in dopamine levels.

The manipulations of dopamine receptors and transporters shown in Figures 4, 5, and 6 support the idea that olfactory stimulation modulates synaptic transmission from OFF bipolar cells by reducing dopamine levels and D_1_ dopamine receptor activity.

### Dopamine Enhances Calcium Currents Controlling Neurotransmission

What are the cellular mechanisms by which dopamine modulates the visual signal transmitted to the inner retina? In the outer retina of fish and mammals, dopamine acts through D_1_ receptors to uncouple horizontal cells providing negative feedback to the synaptic terminals of photoreceptors (Dowling, 1991), but this seems an unlikely mechanism for the selective modulation of transmission through OFF bipolar cells given that these diverge from the ON pathway downstream of photoreceptor output (Schiller et al., 1986). We therefore investigated the possibility that dopamine might also act directly on bipolar cells to modulate synaptic calcium signals.

**Figure 7:**
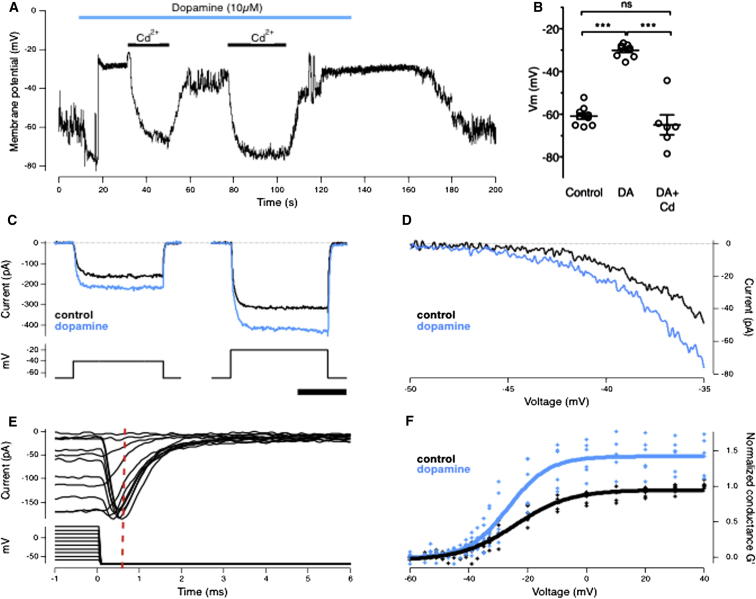
Dopamine Potentiates Voltage-Gated Calcium Channels in Bipolar Cells (A) Voltage recording from an isolated Mb1 bipolar cell. Application of 10 μM dopamine depolarized the cell to approximately −30 mV and this effect was blocked by 100 μM Cd^2+^. Both effects were reversible. (B) Averaged results from n = 9 experiments similar to (A). Dopamine depolarized the membrane potential by 30.7 ± 1.5 mV. (C) Voltage-clamp recordings of the Ca^2+^ current from the terminal of bipolar cells. Example current traces evoked by steps from −70 mV to −40 mV (left) and −20 mV (right) from the same cell, before (black) and after (blue) 10 μM dopamine administration. Scale bar represents 25 ms. (D) The current-voltage relation from −50 to −35 mV for the cell shown in (C). (E) To measure the activation range and peak conductance of the Ca^2+^ currents, the amplitude of the tail current was measured 0.5 ms after the voltage step returned to −70 mV (dashed red line); only increments of 10 mV are shown for clarity. (F) Average steady state activation curves for n = 6 cells, before (black) and after (blue) 10 μM dopamine administration. The data for each cell was normalized to *I*_*max*_ in control. Boltzmann fits (thick lines) are to the population data, giving a *V*_*1/2*_ and *k* of −24.2 ± 0.4 mV and 9.2 ± 0.3 for control and −26.5 ± 0.4 mV and 6.9 ± 0.4 after dopamine administration. The peak conductance increased by a factor of 1.44 ± 0.11. See also Figure S4.

Mixed rod-cone (Mb1) bipolar cells from the retina of goldfish were isolated for electrophysiological recording (Burrone and Lagnado, 1997). In these neurons, voltage-dependent calcium channels are L-type and localized to the synaptic terminal (Burrone and Lagnado, 1997). In current-clamp configuration, using a standard intracellular solution, addition of 10 μM dopamine depolarized bipolar cells by an average of 30.7 ± 1.5 mV, indicating activation of a net inward current (n = 9; Figures 7A and 7B). The depolarization was completely reversed by blocking voltage-dependent Ca^2+^ channels with 100 μM cadmium (Catterall et al., 2003), indicating that dopamine potentiates the calcium conductance (n = 6).

The Mb1 bipolar cell stands out in a preparation of dissociated retinal neurons because of its large terminal. Of the bipolar cells with small terminals, OFF outnumber ON by 3:1 (Odermatt et al., 2012). We also made recordings from the cell bodies of bipolar cell with small terminals, and in all three cases dopamine caused a depolarization of ∼15 mV. It therefore seems very likely that dopamine also acts to enhance calcium currents in OFF bipolar cells. Heidelberger and Matthews (1994) also observed that dopamine potentiated calcium influx in all morphological types of bipolar cell that they tested.

To investigate the actions of dopamine on the calcium conductance more directly, we made voltage-clamp recordings using an intracellular solution designed to block potassium channels. Figure 7C shows the calcium currents elicited by voltage steps from −70 mV to −40 mV and −20 mV in a single cell, and Figure 7D shows an example of the current-voltage relation around the threshold for activation of the calcium current, approximately −43 mV (Burrone and Lagnado, 1997). To quantify changes in the calcium conductance over a number of cells, we measured the amplitude of the tail current 0.5 ms after a voltage step returning to −70 mV (dashed red line in Figure 7E). Averaged conductance-voltage (*G-V*) relations before and after addition of 10 μM dopamine are shown in Figure 7F, with conductance values normalized to the maximum in the absence of dopamine (n = 6 cells). The *G-V* relation could be described by a Boltzmann function (see Experimental Procedures). Addition of 10 μM dopamine increased *G*′_*max*_ by 44% ± 11% and shifted *V*_*1/2*_ from −14.2 ± 0.4 mV to −16.5 ± 0.4 mV (Figure 7F, p = 0.002).

The 2.3 mV shift in *V*_*1/2*_ to lower membrane potentials is significant in the context of the voltage signals that bipolar cells generate in response to light (Baden et al., 2011), which are just a few millivolts in amplitude and span the voltage range at which L-type calcium channels begin to activate. Around this threshold, dopamine potentiated presynaptic calcium currents by a factor averaging 1.9 (Figure 7F). These results demonstrate that dopamine can act directly on bipolar cells to increase the magnitude of the presynaptic Ca^2+^ current that controls transmission of the visual signal. It seems likely that this action makes a significant contribution to the profound increase in the gain of luminance signals observed in vivo in the presence of the dopamine receptor agonist ADTN (Figure 4), as well as the decrease in gain in the presence of the antagonist SCH 23390 (Figure 5).

If an olfactory stimulus acts to lower dopamine levels and therefore inhibits activation of presynaptic calcium channels, one might expect to observe a decrease in the basal calcium concentration in bipolar cells in darkness, with this effect being most obvious in OFF cells resting at more depolarized potentials. We therefore compared resting SyGCaMP2 signals in BC terminals before and after the bath application of methionine (233 ON and 211 OFF from nine fish; Figure S4). Methionine induced a statistically significant reduction in SyGCaMP2 fluorescence in OFF terminals (median = −10.9%, p < 0.01) but not ON (median = −0.2%, not significant), providing further support for the idea that inhibition of presynaptic calcium channels is one of the mechanisms by which an olfactory stimulus reduces the gain of signaling through OFF bipolar cells.

## Discussion

The vertebrate retina receives centrifugal input from a variety of brain regions, depending on the species (Behrens and Wagner, 2004). The olfacto-retinal circuit in fish is a good example of such cross-modal interactions and provides the opportunity to investigate the cellular mechanisms that regulate processing in the early visual system. By monitoring calcium signals in vivo, we find that an olfactory stimulus reduces the gain with which changes in luminance or temporal contrast are transmitted through the OFF pathway, while also increasing sensitivity at lower light levels (Figures 1, 2, and 3). The results demonstrate that the calcium signal controlling neurotransmission from bipolar cells is a key site for regulating the flow of the visual information. The observed modulation of presynaptic calcium responses is likely to contribute to the increase in luminance sensitivity observed behaviorally when the ORC circuit is activated (Maaswinkel and Li, 2003, Huang et al., 2005).

The chemical signal coordinating these changes in retinal performance has been suggested to be a reduction in dopamine release. Strong evidence for this idea is provided by the demonstration that a blocker of dopamine release and reuptake suppresses the change in synaptic gain and sensitivity normally caused by an olfactory stimulus (Figure 6). Manipulations of dopamine receptor activity in vivo are also consistent with this mechanism (Figures 4, 5, and 6) and, in particular, for a key role of D_1_ receptors (Figures 5B and 5D). Finally, we demonstrate that dopamine regulates the activity of voltage-dependent calcium channels in the synaptic terminals of bipolar cells, providing a direct mechanism for regulating the gain of the visual signal (Figure 7). Of course, these results do not rule out the possibility that there are other sites at which ORC also regulates the retinal circuit.

### Olfactory Stimuli Act Primarily on the OFF Pathway

**Figure 8:**
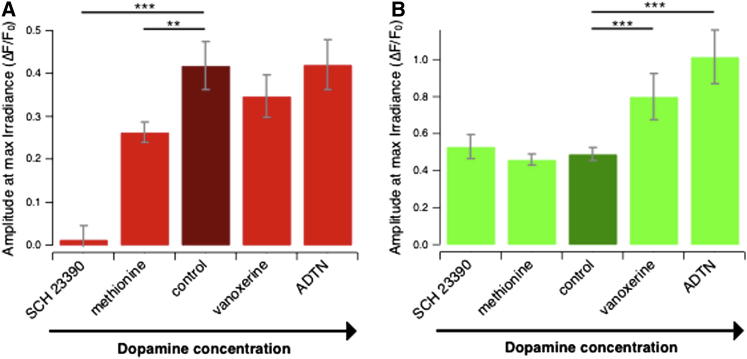
A Comparison of Average Synaptic Gain in ON and OFF Bipolar Cells under a Variety of Conditions that Modulate Dopamine Signaling in the Retina Bar graphs showing the average amplitude (±SEM) of the SyGCaMP2 response to the brightest light in OFF (A) and ON (B) bipolar cells. The various conditions are ordered according to the expected level of dopamine receptor activation. Note that, under control conditions (9:00–11:00 a.m.), synapses of OFF bipolar cells responded to light with maximal gain equivalent to that measured in the presence of the dopamine receptor agonist ADTN. In contrast, under control conditions synapses of ON bipolar cells operated at minimum gain equivalent to that measured in the presence of the dopamine receptor agonist SCH 23390.

An overview of changes in the amplitude of the calcium signal through ON and OFF bipolar cell terminals is shown in Figure 8. The response is quantified as the relative change in SyGCaMP2 fluorescence caused by a bright step of light applied from darkness, and the various experimental conditions are ordered according to the expected level of dopamine activity, with the measurement in 100 nM of the D_1_ dopamine receptor antagonist SCH 23390 at one extreme and in 200 nM of the agonist ADTN at the other. This comparison reveals a fundamental difference in the sensitivity of the ON and OFF pathways to changes in retinal dopamine levels. Under control conditions, luminance signaling through the OFF pathway is operating at its maximum gain (i.e., similar to that measured in ADTN), whereas signaling through the ON pathway is operating at its minimum gain (measured in SCH 23390). Thus, although an olfactory stimulus that results in decreased dopamine levels may be expected to decrease the gain of signals through the OFF pathway (Figures 1 and 8A), it is not expected to suppress synaptic calcium signals in ON bipolar cells (Figures 1 and 8B). It appears that the ON and OFF pathways have different sensitivities to dopamine.

The mechanism for the differential effects of dopamine on the ON and OFF pathways is still not clear. ON and OFF bipolar cells both express D_1_ receptors but not D_2_ (Fan and Yazulla, 2005, Yu and Li, 2005). D_1_ receptors act through *G*_*s*_ proteins which couple to adenylyl cyclase to increase cAMP and direct activation of adenylyl cyclase by forskolin also increases bipolar Ca^2+^ responses (Heidelberger and Matthews, 1994). A possible explanation for the contrasting effect in ON versus OFF could be differential sensitivity of the Cav channels to cAMP that may reflect which Cav channels underlie the response (Pan et al., 2001, Logiudice et al., 2006). An alternative possibility is that the ON and OFF channels are regulated by a second neuromodulator, which interacts with dopamine pathways. For instance, Iuvone and Gan (1995) have demonstrated that activation of MT2 melatonin receptors antagonizes signaling through D_1_ dopamine receptors in bipolar cells by inhibiting cAMP synthesis through a *G*_*i*_ protein, and Wiechmann and Sherry (2012) have found that MT2 melatonin receptors are localized to OFF but not ON bipolar cells in *Xenopus laevis*. The fast decrease in melatonin concentration that occurs after dawn might therefore act to enhance selectively the sensitivity of OFF bipolar cells to variations in dopamine levels.

We did see a small population of ON bipolar cell terminals (∼9%) strongly potentiated by olfactory stimulation (Figures 1I, S1A, and S1B). Might this reflect differences in the mechanism by which glutamate released from photoreceptors act on different types of ON bipolar cells? In zebrafish, some ON bipolar cells respond through metabotropic glutamate receptors and others through a glutamate transporter with a large chloride conductance (Connaughton and Nelson, 2000, Nelson and Connaughton, 2004). Although the former mechanism predominates in mixed rod-cone bipolar cells with large terminals, the latter occurs in cone bipolar cells with smaller terminals. We tested, therefore, if there was any relationship between the size of ON terminals and their response to methionine, but did not find any; i.e., the size distribution of ON terminals responding to methionine was very similar to those that did not, both varying between ∼0.6 μm and ∼5 μm in radius. We also investigated whether there might be any relation between the location of ON terminals within the IPL and their response to methionine and again there was not. As a consequence, at present we do not have elements to consider this as a separate subpopulation of ON bipolar cells.

### Olfactory Stimuli Modulate Retinal Gain and Sensitivity

Our results are consistent with the hypothesis that odor stimulation reduces the conductance and shifts the *V*_*1/2*_ of Cav channels in bipolar terminals, with dopamine being the key mediator. This mechanism is able to explain several of the observed effects of olfactory stimulation on the transmission of visual information through bipolar cells. For example, the decreased conductance and shift in the activation of the calcium channels will lower Ca^2+^ influx for small depolarizations such as low contrasts, nevertheless larger depolarizations, such as high contrasts, will still be effective. This manifests in a more nonlinear contrast response function (Figure 2B) with greater sensitivity for higher contrasts. The decrease in the Ca^2+^ channel maximum conductance also explains the lower gain seen at maximum luminance (Figure 1D). This highlights the presynaptic terminal of bipolar cells as a key site for regulating the transmission of visual signals through the retina.

As well as the dramatic gain reduction, the OFF pathway also becomes more sensitive to dimmer light. As the expected effects of reduced dopamine will shift the Cav activation to more depolarized potentials, it is unlikely to explain the increased luminance sensitivity. However, D_1_ receptors do enhance glutamate-gated ionic channels in OFF bipolar cells (Maguire and Werblin, 1994). When D_1_ receptors are activated, ionotropic glutamate receptors generate enhanced current that will result in OFF bipolar cells being less sensitive to small decreases in glutamate concentration; a similar phenomenon has been described in horizontal cells (Knapp and Dowling, 1987).

### The Physiological Significance of Olfactory Modulation in the Visual System

The olfacto-retinal circuit endows the vertebrate visual system with the ability to quickly reduce the gain and increase the sensitivity of the retina in the presence of food, independently of changes in mean luminance. A behavior that is likely to be related to this process has recently been described by Stephenson et al. (2011), who found that zebrafish show a preference for darker areas in their environment when background levels of light are low, and brighter areas when background light levels are high. An olfactory stimulus applied in low background would then mimick the effects of light adaptation by encouraging fish to explore brighter areas. The reduction in gain of bipolar cell synapses transmitting the visual signal to the inner retina (Figure 1), as well as the increase in sensitivity to high contrast (Figure 2), is likely to be one of the mechanisms by which an olfactory stimulus allows the visual system of the zebrafish to operate in brighter areas.

In the future, it will be interesting to investigate the behavioral consequences of a selective decrease in gain of the OFF pathway. Certainly it would be expected to help the retina avoid saturation under bright conditions, but then so would a decrease in gain through the ON pathway. A possible explanation for the selective control of the OFF pathway might lie in the recent study of Ratliff et al. (2010) who asked why OFF RGCs are so much more numerous than ONs in most retinas (including zebrafish). They found that natural scenes contain an excess of negative spatial contrasts over positive, leading to the suggestion that the excess of OFF RGCs is a structural adaptation of the retina to the excess of darkness in natural scenes. In zebrafish, OFF bipolar cells outnumber ONs by a ratio of 3:1 (Odermatt et al., 2012), so it may be that a decrease in the gain of the OFF pathway is the most important adaptation required to process negative contrasts at higher mean light levels. Another possible explanation could be linked to the differential role proposed by Burgess et al. (2010) for the two systems, being the ON pathway mainly involved in appetitive behaviors and the OFF pathway more implicated in escape responses. In this perspective, the observed food odor-induced inhibition of the OFF would suppress escape responses, thus favoring appetitive behaviors.

The “re-tuning” of retinal processing by a food-related olfactory stimulus is likely to be relevant to different aspects of zebrafish behavior, but especially hunting and prey-capture. The observed increase in the gain of the ON channel relative to the OFF is expected to make the retina more sensitive to regions of positive contrast, such as bright spots appearing when sunlight reflects off small prey. Bright spots are an effective stimulus for eliciting prey-capture behavior in a “virtual reality” assay (Bianco et al., 2011), and this may provide an experimental context in which to study the behavioral consequences of olfactory-visual integration.

## Experimental Procedures

### Transgenic Zebrafish

All procedures were carried out according to the UK Animals (Scientific Procedures) Act 1986 and approved by the UK Home Office. We made transgenic zebrafish (*Danio rerio*) expressing the synaptically localized fluorescent calcium reporter SyGCaMP2.0 under the ribeye-A promoter, as in Dreosti et al. (2009) and Odermatt et al. (2012), or the calcium reporter GCaMP3.5 under the eno2 promoter, as in Bai et al. (2007). SyGCaMP2 and GCaMP3.5 zebrafish were kept at a 14:10 hr light:dark cycle and bred naturally. Larvae were grown in 200 μM 1-phenyl-2-thiourea (Sigma) from 28 hr postfertilization to inhibit melanin formation (Karlsson et al., 2001). Forty-eight fish were used in these experiments.

### Two-Photon Imaging In Vivo

Whole zebrafish larvae (8–11 days postfertilization [dpf]) were immobilized in 2.5% low melting point agarose (Biogene) on a glass coverslip and submersed in E2 embryo medium (Nusslein-Volhard and Dahm, 2001). Bipolar cell terminals were imaged in vivo using a custom-built two-photon microscope equipped with a mode-locked Chameleon titanium-sapphire laser tuned to 915 nm (Coherent) with an Olympus LUMPlanFI 40× water immersion objective (N.A. 0.8). Emitted fluorescence was captured through both the objective and a substage oil condenser, filtered through a HQ 520/60 m-2P GFP emission filter (Chroma Technology) and detected by a set of photo-multiplier tubes (Hamamatsu). Scanning and image acquisition were controlled under ScanImage v.3.6 software (Pologruto et al., 2003). All recordings were performed between 9:00 and 11:00 a.m., except when otherwise stated. Full-field light stimuli were delivered by amber LEDs (Luxeon), 590 nm band-passed ±10 nm, and controlled in Igor Pro 4.01 (WaveMetrics) and time locked to image acquisition.

### Stimulation Protocols

Bipolar cell terminal responses were analyzed in terms of light sensitivity (irradiance), contrast sensitivity, and frequency sensitivity. Retinal ganglion cells were analyzed in term of contrast sensitivity, only. For this purpose we designed three different protocols of stimulation.

Luminance (irradiance) sensitivity was assessed by stimulating the dark-adapted fish with a series of flashes (4 × 3 s flashes at 6 s intervals) at nine different light intensities, ranging between 11 pW/mm^2^ and 110 nW/mm^2^ with 0.5 log unit steps. The sequence of light intensities was randomized to reduce habituation artifacts in the recordings. Maximum light intensity, 110 nW/mm^2^, is equivalent to 3.3 × 10^11^ photons/mm^2^ × s^−1^.

Contrast sensitivity was assessed by stimulating the dark-adapted fish with a series of 10 s light oscillations at 5 Hz around a constant light level (55 nW/mm^2^) at 10 different levels of contrast, ranging from 10% to 100% of the constant light level.

Finally, frequency sensitivity was assessed by stimulating the dark-adapted fish with a series of 10 s light oscillations around a constant light level (55 nW/mm^2^) at 90% contrast at 14 different frequencies, ranging from 0.2 to 25 Hz.

Image sequences were acquired at 10 Hz (256 × 100 pixels per frame, 1 ms per line) for the irradiance and contrast experiments and at 40 Hz (256 × 25 pixels per frame, 1 ms per line) for frequency experiments.

### Drugs

The stimulation of the olfactory bulb was obtained by bath application of the amino acid methionine (Sigma) 1 mM, as in Maaswinkel and Li (2003).

To manipulate dopamine signaling in the retina we injected neuroactive drugs into the eye. Final concentrations of the drugs were calculated by diluting the injected concentration into the free volume of the eye. The volume of a typical 9 dpf old zebrafish eye was assessed by three-dimensional reconstruction of the eye chamber and the lens through two-photon microscopy scanning. The final volume was estimated as the difference between the total eye volume and the volume of the lens core. We calculated a total free volume of ∼500 μm^3^. Given a typical injected volume of 10 μl, the final dilution factor can be approximated to 1:50.

Dopamine receptors were activated by injection of the long-lasting dopamine receptor ligand [3H] 2-amino-6,7-dihydroxy 1,2,3,4-tetrahydronapthalene (ADTN) (Sigma) 10 μM, as in Li and Dowling (2000b). Dopamine action on postsynaptic targets was prevented by injection of the strong dopamine D_1_ receptor antagonist SCH 23390 (Sigma) 2 nM, as in Huang et al. (2005) or the selective dopamine D_2_ receptor antagonist sulpiride (Sigma), as in Lin and Yazulla (1994) and Mora-Ferrer and Gangluff (2000). Finally, the level of dopamine in the circuit was frozen by injection of the dopamine release and reuptake inhibitor vanoxerine (Santa Cruz Biotechnology) 2 μM, as in Schlicker et al. (1996).

### Analysis of Two-Photon Imaging Data

Preprocessing was carried out in Image J (National Institutes of Health) and consisted of stack registration and Kalman stack filter denoising (filter gain = 0.6). Regions of interest (ROI) extraction, background subtraction, and brightness normalization (ΔF/F_0_) were performed in Igor Pro 6.2 and facilitated by SARFIA analysis routines (Dorostkar et al., 2010). Fluorescence traces were then sorted and analyzed by custom-made scripts and NeuroMatic. The detection of active ROI in the IPL was based on the thresholding of the Laplacian Transform of the two-photon recordings. In this way, responding bipolar cell terminals and active areas of the ganglion cell dendrites were identified in ribeye::SyGCamp2 and eno2::GCamp3.5 fish, respectively.

The responses to light of bipolar cell terminals and retinal ganglion cell dendrites were characterized according to their response amplitude, i.e., the variation in fluorescence during stimulation in comparison to baseline (ΔF/F_0_). Responses to light were plotted in full, as in Figure 1B, left, or in stimulus versus amplitude plots (e.g., Figure 1B, right). In the case of traces representing single terminals (e.g., Figure 1B), the error curve (gray shadow in Figure 1B) represents the SEM of the four trials employed to assess the terminal responsiveness (see Stimulation Protocols). In the case of traces representing whole populations of terminals (e.g., Figure 1D), the error curve represents the standard error of all the responses employed to generate the final average. As described in the stimulation protocols section, a stimulus could be light intensity, contrast, or frequency. Intensity versus amplitude plots were obtained by averaging amplitude values over 300 ms long time windows around the maximum response occurring during the stimulation time (e.g., Figure 1B, right). Contrast versus amplitude and frequency versus amplitude plots were obtained by averaging amplitude values over the whole stimulation period (e.g., Figures 2B and S2D, respectively).

The intensity versus amplitude plots were fitted with Hill curves, in the form *A* = *I* ^*h*^/*I* ^*h*^ + *I*_*1/2*_^*h*^, *A* being the response amplitude, *I* the stimulation intensity, *h* the Hill coefficient, and *I*_*1/2*_ the sensitivity at half maximum, i.e., the stimulation intensity that elicits half of the maximum response. *I*_*1/2*_ has been used as a metric for the sensitivity of each intensity versus amplitude curve. Contrast versus amplitude plots were fitted with power functions, in the form *A* = *k* × *C*^*α*^ being *A* the response amplitude, *k* a constant, *C* the stimulation contrast, and α the power exponent.

The sensitivity shift induced by olfactory stimulation for each individual terminal (e.g., Figure 1F) was measured by comparing the values of the lowest light intensity eliciting a statistically significant response before and after methionine administration. The statistical significance of a response was assessed by comparing (t test) the average calcium level during light stimulation with a threshold defined as three times the SD of a baseline epoch.

The effect of drugs or of olfactory stimulation has been also described in terms of percentage variation in the amplitude of response to the maximum irradiance stimulus (e.g., Figure 1E).

### Electrophysiology

Goldfish (*Carassius auratus*) were dark-adapted for 1 hr and killed by decapitation followed immediately by destruction of the brain and spinal cord under Schedule 1 of the UK Animals (Scientific Procedures) Act 1986. Depolarizing bipolar cells were isolated from the retina of goldfish by enzymatic digestion, using methods described by Burrone and Lagnado (1997). The standard Ringer solution contained the following: 110 mM NaCl, 2.5 mM CaCl_2_, 2.5 mM KCl, 1 mM MgCl_2_, 10 mM glucose, and 10 mM HEPES (260 mOsmol l-1, pH 7.3). The solution in the patch pipette to record voltage membrane in current-clamp experiments contained: 110 mM K-gluconate, 4 mM MgCl_2_, 3 mM Na_2_ATP, 1 mM Na_2_GTP, 0.5 mM EGTA, 20 mM HEPES, and 10 mM Na-phosphocreatine (260 mOsmol l-1, pH 7.2). To isolate Ca^2+^ channel currents, the intracellular solution contained 110 mM Cs-gluconate, 4 mM MgCl_2_, 3 mM Na_2_ATP, 1 mM Na_2_GTP, 10 mM tetraethylammonium chloride, 20 mM HEPES, 0.5 mM EGTA, and 10 mM Na-phosphocreatine (260 mOsmol l-1, pH 7.2). Room temperature solutions were superfused via a fast perfusion system (VC8-S; ALA Scientific). Patch electrodes with 5–7 MΩ tip resistance were pulled from fire-polished borosilicate glass capillary tubes using a micropipette puller (Sutter Instrument). The series resistance was typically 8–15 MΩ on rupturing the patch. Holding current in current-clamp configuration was 0 pA. Voltage-clamp and current-clamp recordings were made in synaptic terminals. In voltage-clamp experiments, the membrane potential was held at −60 mV, and stimuli were delivered by stepping the membrane potential to −10 mV. To construct *G/V* plots the tail current amplitude measured 0.5 ms after returning to −70 mV was plotted against the preceding voltage step. The voltage dependence of activation was determined from normalized conductance versus voltage curves, which were fitted according to the Boltzmann function:

$$G^{\prime }=\frac{G^{\prime }\max}{1+\exp\left(\frac{V-V_{1/2}}{k}\right)},$$

where *G*′ is the normalized conductance, *V*_*1/2*_ is the membrane potential at which activation is half-maximal, and *k* is the slope factor.

Signals were recorded using an Axopatch 200A amplifier (Molecular Devices), interfaced with an ITC-16 (HEKA) and controlled with Pulse Control 4.3 running under Igor Pro 5 (Wavemetrics). Data were given as the mean ± SEM.
