# Supplementary material for: Olfactory Stimulation Selectively Modulates the OFF Pathway in the Retina of Zebrafish
Source: Neuron. 2013 Jul 10;79(1):97–110. doi: 10.1016/j.neuron.2013.05.001 (PMC3710973; doi:10.1016/j.neuron.2013.05.001)
Supplement: Document S1. Figures S1–S4 [file mmc1.pdf]

Neuron, Volume *79*

Supplemental Information

Olfactory Stimulation Selectively Modulates

the OFF Pathway in the Retina of Zebrafish

Federico Esposti, Jamie Johnston, Juliana M. Rosa, Kin-Mei Leung, and Leon Lagnado

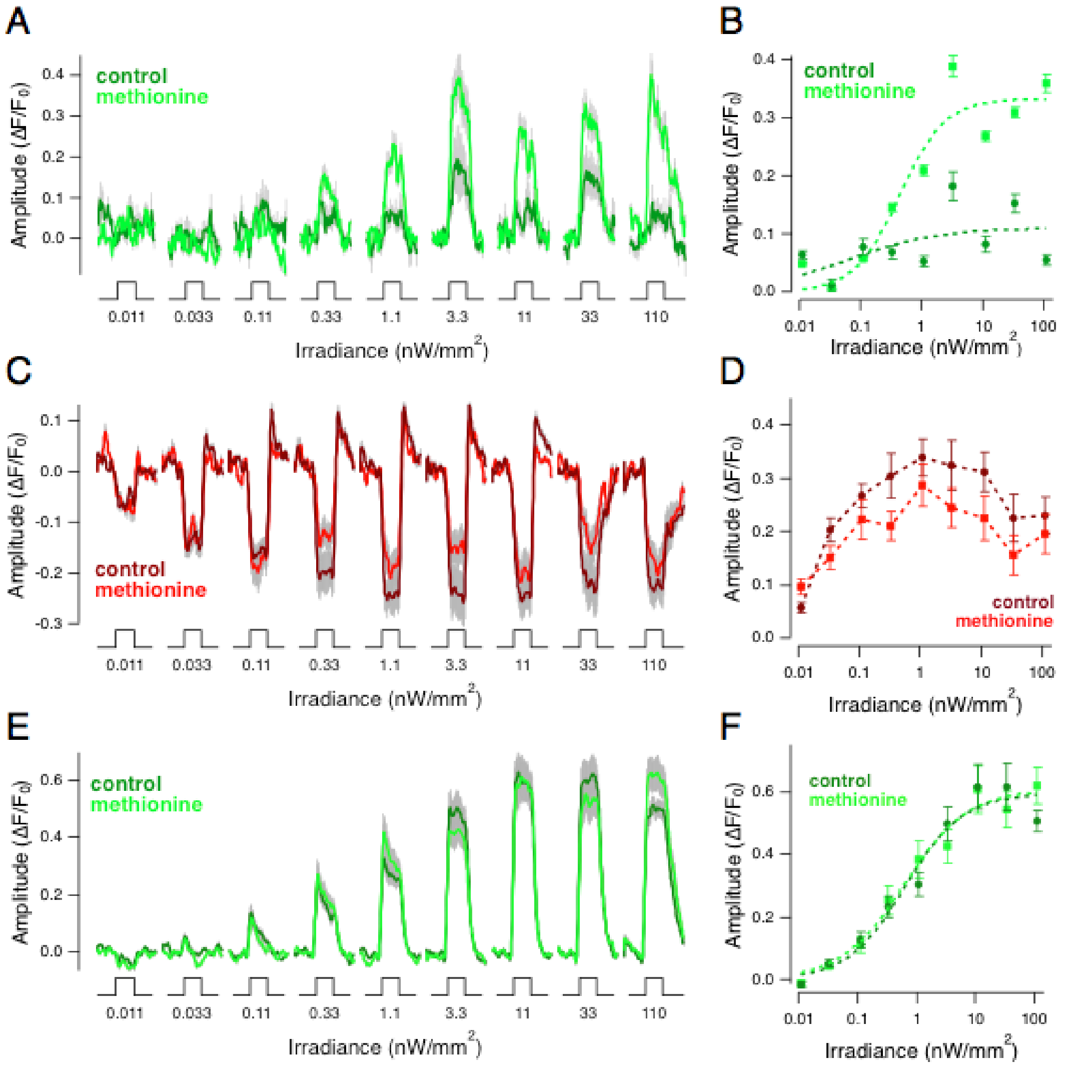

Figure S1. Odour stimulation potentiates a subpopulation of ON bipolar cell terminals (A-B). The effect of olfactory stimulation in the afternoon (C-F). Related to Figure 1

(A) Averaged responses of the subpopulation of ON bipolar cell terminals (7 fish, *n* = 14 terminals) potentiated by odour stimulation (arrowed in Figure 1I) to irradiance protocol, as in Figure 1. Control responses in dark green responses after olfactory stimulation in light green. SEM indicated in grey.

(B) Plot of intensity vs. response amplitude averaged from the same populations of ON terminals shown in (A). Dotted lines are fits of the Hill function (see main text, *Experimental Procedures*) with h = 0.77 and $I_{1/2}$ = 0.13 nW/mm^2 in control and h = 1.05 and $I_{1/2}$ = 0.48 nW/mm^2 in methionine. Note how after stimulation the terminals reach h and $I_{1/2}$ values similar to the rest of the ON population (cf. Figure 1H, h = 1.14, $I_{1/2}$ = 0.60 nW/mm^2 in methionine).

(C-D) Averaged responses and intensity vs. response amplitude plot of 72 OFF bipolar cell terminals from 5 fish (8-11 dpf) to the same protocol shown in Figure 1, before (dark red) and after (light red) bath administration of 1mM methionine. These recording were performed between 12 and 3.30 pm. OFF bipolar cell terminals provided best responses at 1.1 nW/mm^2.

At this light intensity, methionine induced an average amplitude reduction of 14.7 ± 3.4%, significantly smaller than the 36.6 ± 8.16% observed in the early morning (p = 0.02).

(E-F) Averaged responses and intensity vs. response amplitude plot of 50 ON bipolar cell terminals from 5 fish (8-11 dpf) to the same protocol shown in Figure 1, before (dark green) and after (light green) bath administration of 1mM methionine. These recordings were performed between 12 and 3.30 pm. ON bipolar cell terminals provided best responses at 110 nW/mm^2, similarly to early morning recordings. No effect of methionine administration could be recorded.

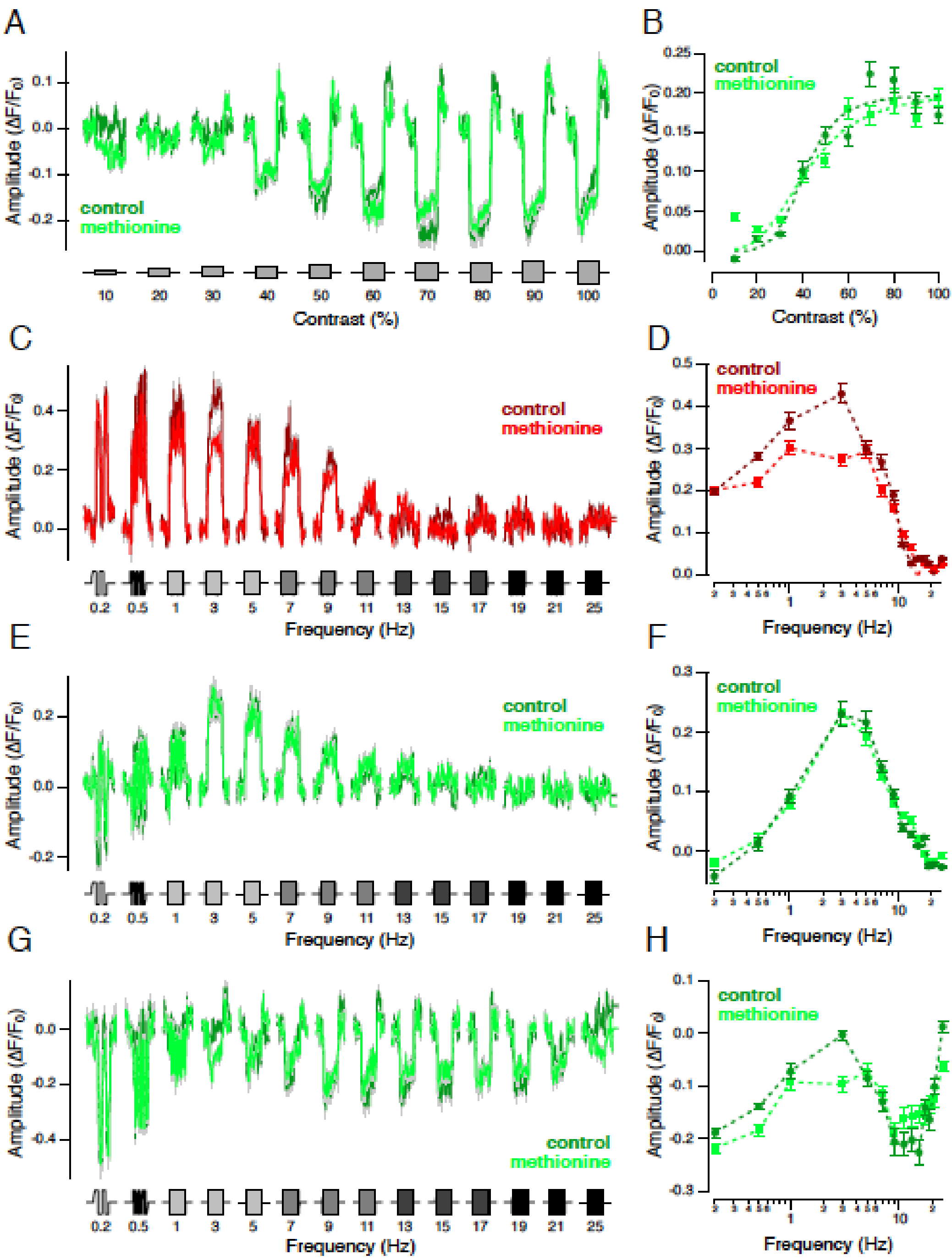

Figure S2. The effect of odour stimulation on contrast and frequency responses. Related to Figure 2

(A) Averaged responses of contrast-inhibited ON bipolar cell terminals (5 fish, *n* = 33 terminals) to temporal contrast protocol (cf. Figure 2C-D). Control responses in dark green and responses in methionine in light green. SEM indicated in grey. Interestingly, by surveying the responses to variations in temporal contrast of a large population of ON bipolar cell, we found a surprisingly high number of synaptic terminals (46%) responding with a decrease in mean SyGCaMP2 fluorescence.

(B) Plot of contrast vs. response amplitude averaged from the same population of ON terminals shown in (A). Dotted lines are fits of a Hill function with h = 5.51, $I_{1/2}$ = 40.93% in control and h = 3.43, $I_{1/2}$ = 41.82% in methionine. No clear effect of methionine administration could be recorded.

(C) Averaged responses of 54 OFF bipolar cell terminals to stimuli of frequencies between 0.2 and 25 Hz (90% contrast). Control responses in dark red and responses in methionine in light red. SEM indicated in grey. Please note that the dynamics of our reporter, SyGCaMP2, didn't allow any quantitative evaluation of the behaviour over 10 Hz.

(D) Plot of response amplitude as a function of frequency averaged from the same population of OFF terminals shown in (C). The suppression of synaptic responses by methionine was evident at frequencies of 5 Hz and below.

(E) Averaged responses of 42 ON bipolar cell terminals from 6 fish to stimuli of frequencies between 0.2 and 25 Hz (90% contrast). Control responses in dark green and responses in methionine in light green. SEM indicated in grey.

(F) Plot of frequency vs. response amplitude averaged from the same population of ON terminals shown in (E). Methionine did not significantly alter the synaptic calcium signal at any frequency.

(G) Averaged responses of contrast-inhibited ON bipolar cell terminals from 5 fish to frequency protocol. Control responses in dark green and responses in methionine in light green. SEM indicated in grey.

(H) Plot of frequency vs. response amplitude averaged from the same population of ON terminals shown in (G). Dotted lines are interpolation of the data.

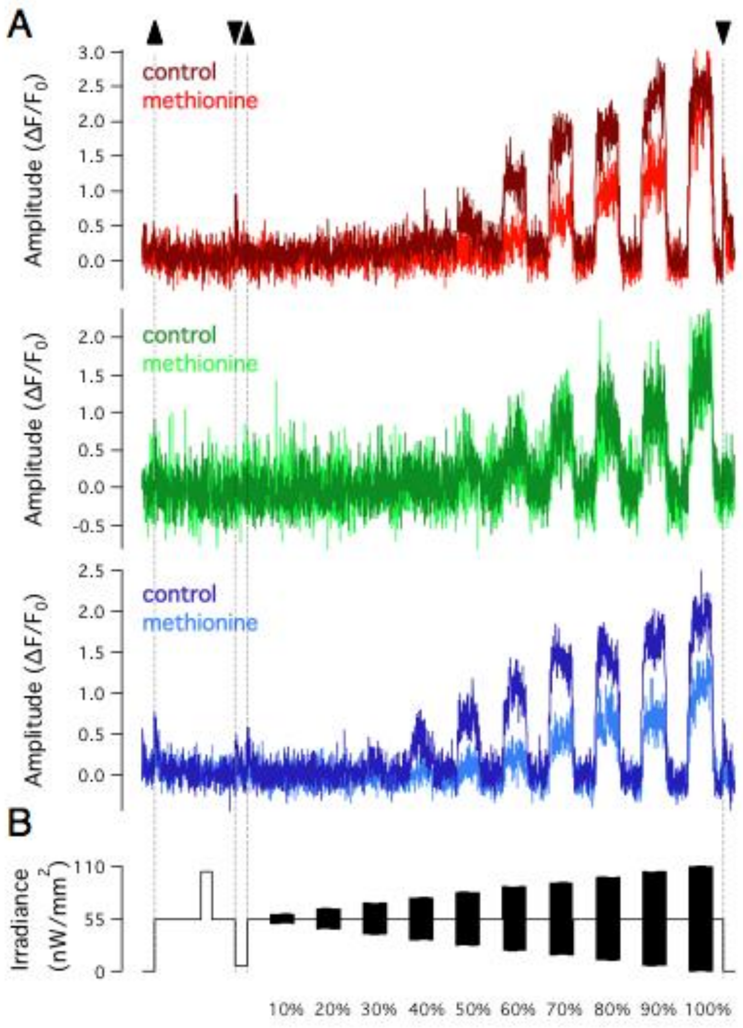

Figure S3. Retinal Ganglion Cell dendritic responses can be classified according to their response to mean light level changes. Related to Figure 3

The polarity of the responses of individual retinal ganglion cells (Figure 3A) can be classified as OFF, ON or ON-OFF depending on the response to increments (upward arrows) or decrements (downward arrows) of the mean light level stimulating the fish's retina.

(A) Three examples of individual eno2::GCaMP3.5 responses, before and after methionine bath administration. Top: OFF, responding to downward deflections of the mean light level. Middle: ON, responding to upward deflections of the mean light level. Bottom: ON-OFF, responding to both downward and upward deflections of the mean light level.

(B) Protocol employed to assess the RGC polarity and response to contrast.

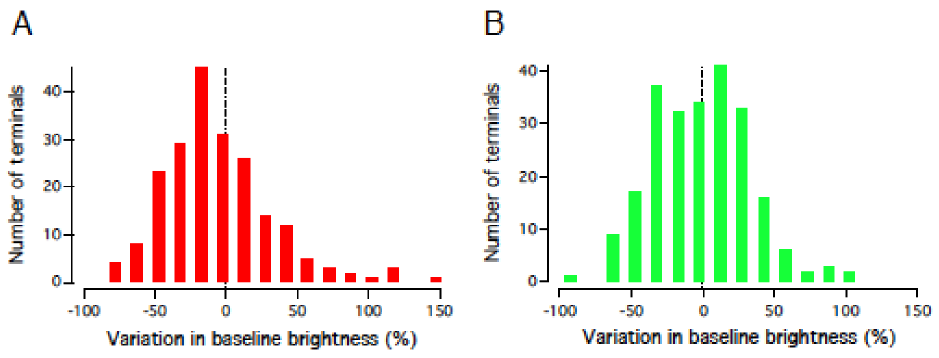

Figure S4. Methionine administration decreases the resting calcium concentration of OFF bipolar cell terminals. Related to Figure 7

To verify the modulation of basal calcium concentration *in vivo*, we tested the variation in resting fluorescence following olfactory stimulation on n = 9 ribeye::SyGCaMP2 fish.

(A) Histogram showing the percentage variation in SyGCaMP2 baseline brightness of 211 OFF terminals from 9 fish, before and after administration of 1mM methionine in the bath. Median decrease -10.9%, significantly different from 0 (Wilcoxon signed rank test, $p < 0.01$).

(B) Histogram showing the percentage variation in SyGCaMP2 baseline brightness of 233 ON terminals from 9 fish, before and after administration of 1mM methionine in the bath. Median variation 0.2% (not statistically different from 0, Wilcoxon signed rank test).
